# Cardiac MRI and pulmonary hypertension-the pressure is on!

**DOI:** 10.1186/1532-429X-16-S1-T6

**Published:** 2014-01-16

**Authors:** Ronald B Williams, Mark Doyle, Moneal Shah, June A Yamrozik, Diane V Thompson, Robert W Biederman

**Affiliations:** 1 Cardiac MRI, Allegheny General Hospital, Pittsburgh, Pennsylvania, USA

## Background

Severe Pulmonary Hypertension (PAH/PH) has high morbidity and mortality. CMR is the 'gold standard' for evaluation of RV structure and function. However, currently CMR has no means to directly measure right heart pressures analogous to the invasive RHC. Hypothesis: RV/LV geometric indices are predicative of right ventricular pressure measured during the RHC.

## Methods

Subjects were eligible for entry if they had documented PAH as defined by WHO Criteria and were referred for a clinically indicated CMR exam where routine 3D metrics and LGE were performed. All images were retrospectively reviewed. The data collected as follows: right and left ventricular RV/LV EDVI and ESVI; RVEF and LVEF; RV anterior and inferior hinge point angle measurements (systole and diastole); LV diameters to determine eccentricity index in diastole and systole; RV hinge point LGE and right heart catheterization: RV and pulmonary arterial (PA) pressures. The hinge point angles were measured at the level of the head of the papillary muscles in the short axis. LGE was determined on the SA LGE images as not present, mild, moderate or severe (zero, 1, 2 or 3). All were related to right heart derived pressures. All patients were analyzed using IBM Statistical Package, PASW, version 18 (Formally SPSS).

## Results

Fifty (50) subjects were analyzed including 37 patients with documented PAH (22 females, 50 ± 22 yrs.; 15 males, 56 ± 23 yrs). A group of 13 non-PAH subjects who underwent routine CMR served as the control group (5 females, 43 ± 3 yrs.; 8 males, 35 ± 13 yrs.). Regarding the 37 PAH patients, 6 did not have a RHC within the pre-specified time (2 months). The remainder had RHC within 2 weeks ± 5 days of the CMR exam. Measurements were included to determine the degree of correlation with RHC data. In univariate analysis, no single parameter predicted PAP with statistical significance and clinical value. However, for RV pressure multivariate analysis showed that combining Right Ventricular Anterior Angle in systole RVAAs) (r = 0.404), Left Ventricular Eccentricity in systole (LVECCs) (r = 0.43) in a linear regression model, better predicts RV pressure than the variables used separately: RV pressure index = [12.34+0.509*RVAAs+9.031*LVECCs] (r = 0.52, p < 0.01). For the controls the RV pressure index was lower than in patients (49 ± 6 vs. 71 ± 15, p < 0.001). Further, LGE grading correlated with the RV pressure, but to a lesser degree (r = 0.49).

## Conclusions

Non-invasive testing has been shown to determine the patient's prognostic future. Herein we show RV angles and systolic eccentricity, when combined in a linear regression model better predicts RV pressure than does LGE extent in the RV hinge point region. This model may provide earlier indicator of risk.

## Funding

Internal.

**Figure 1:**
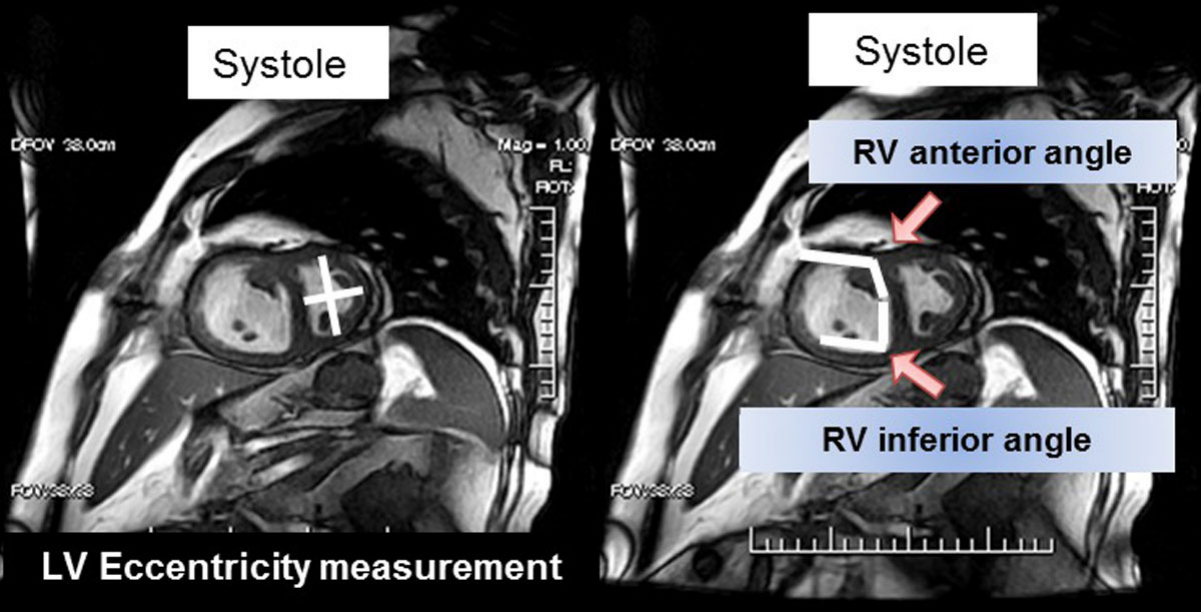
**Eccentricentsmity Measurements/RV angle measure**.

**Figure 2:**
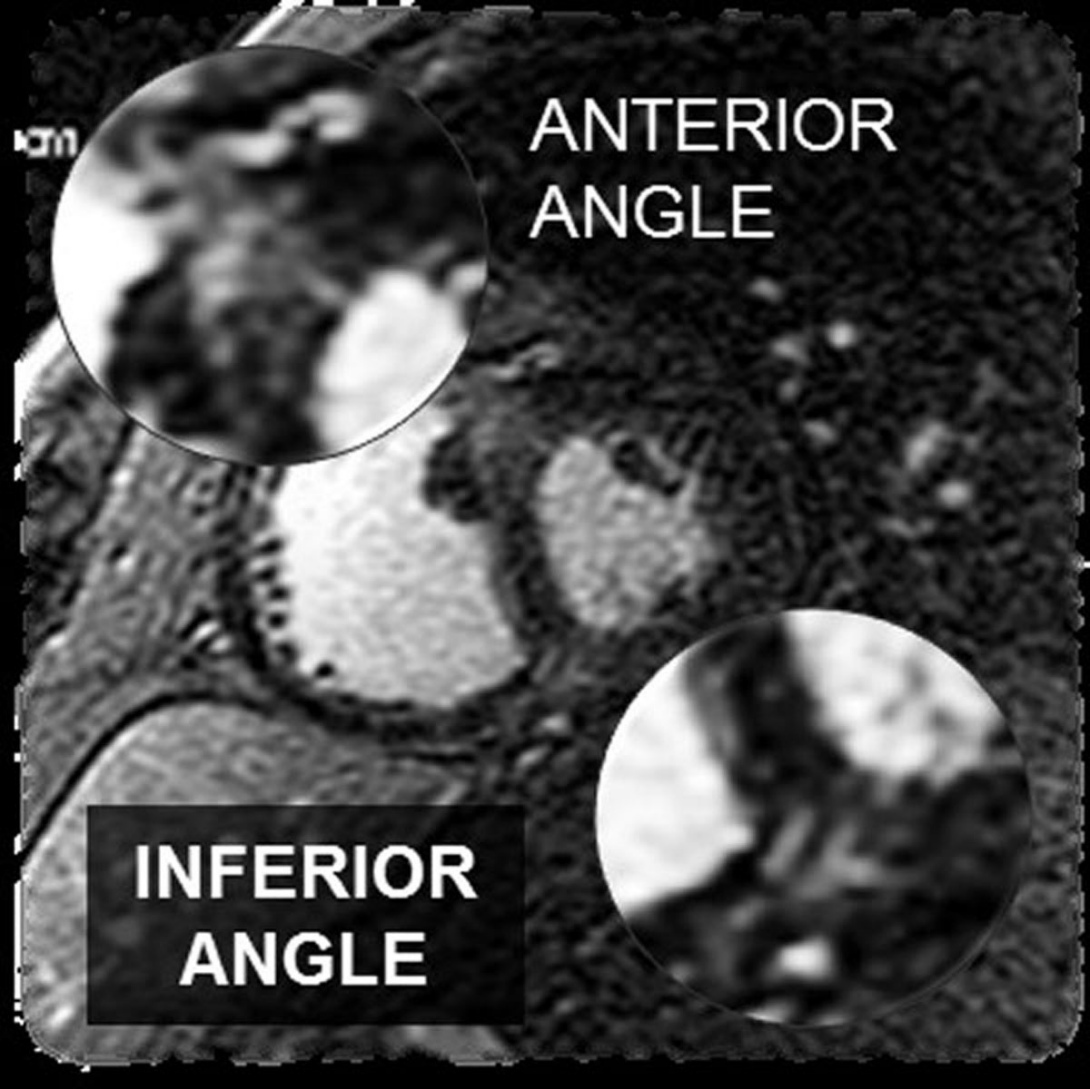
**RV insertion Point LGE**.

